# How Long Halt Ye?

**Published:** 1920-12-11

**Authors:** Richard J. Coles

**Affiliations:** Secretary, King's College Hospital


### THE EDITOR'S LETTER-BOX.

**Correspondence on all subjects is invited, but we cannot in any way be responsible for the opinions expressed by our correspondents, who must give their name and address as a guarantee of good faith, but not necessarily for publication. Correspondents are reminded that brevity of style and conciseness of statement greatly facilitate early insertion.**

### How Long Halt Ye?

*To the Editor of* THE HOSPITAL.

SIR,—I have read with profound interest your leading article in this week's issue of THE HOSPITAL under the title of "How Long Halt Ye?"

With you, most emphatically I refuse to believe either that the voluntary-hospital system is dead, or that what can be achieved in the provinces cannot be achieved in London.

Before I made the suggestion that the 2,010,000 insured persons should be asked to contribute regularly one penny per week, and their employers a similar amount in respect of them, I had reviewed the whole situation, and it was my intention, whilst urging the insured persons not to excuse the *non-insured*, but to appeal to them for regular support in a like manner, so that all salaried and wage earners might be led to fulfil their Christian privilege and obligation to their sick and suffering fellows.

I am constantly told that London is so different from the provinces, because it has not for its hospitals the same local interest. If there is any truth in such a contention, and I am not prepared to believe it, then the sooner we in London set to work to organise and create local and general interest the better.

The possibilities of continuing to maintain our great hospitals in London on the voluntary principle, in my opinion, have not yet been exhausted. What we want to work for is a contribution at least of 2d. per week from everyone earning, whether professionally or otherwise, both men and women, over the age of sixteen. I am unwilling to believe that such a scheme cannot be established. There is but one way to preserve and hand down to posterity the glorious heritage of our voluntary hospitals, and their scientific research and work of mercy and healing, and that is to delay no longer, but for each and every one of us to agree to give at least 2d. per week regularly to support and efficiently maintain these grand institutions. How is this to be accomplished?

In my opinion, in London we must organise, take immediate steps to bestir that spirit of sympathy and willingness which lies latent within us, and this can be done by the whole of the London hospitals making up their minds, in the first instance, to unite, arrange hundreds and, if needs be, thousands of meetings for the purpose of getting face to face with the people and explaining to them the whole situation, and obtaining from them the promise of their support, and of showing them how they can give it; further, to impress upon them that it must be their scheme for their own hospitals, and that they who contribute in this way must control the funds raised, and decide as to how it is to be distributed. The organising of such a number of meetings can only be carried out by enlisting the services of thousands of helpers all over London.

For such a scheme as I have outlined, the employers of every firm, or place of business, whether a factory, a warehouse, a wholesale establishment, an office, or a small retail shop—in other words, *every employee*, no matter what his or her occupation may be—should agree to voluntarily give 2d. per week to help the hospitals, and should request in writing his or her employer to retain that amount when paying the salary or wage each week, for this purpose, and hand same over either weekly, monthly, or quarterly to the treasurer of the fund. The employers should not be behind in this matter; they should not only be willing to assist by retaining, at their employees' request, the amount, but should themselves give 2d. per week in respect of each employee to the Hospital Fund. This method of collecting has been adopted by the employees of the Great Eastern Railway Co., Cambridge District, and the company retain and hand over the amounts. This saves contributors all trouble, is secure, and there is no easier method of collecting such contributions than this.—Yours faithfully,

RICHARD J. COLES,
Secretary, King's College Hospital.

---

*To the Editor of* THE HOSPITAL.

